# Monument to the Late Dr. Beers

**Published:** 1901-02

**Authors:** 


### MONUMENT TO THE LATE DR. BEERS.

THE *Montreal Daily Witness* advocates the erecting a monument to the late Dr. Beers. This has no connection with his reputation as a dentist; indeed, that part of his life-work seems to have been a very secondary consideration, if it has been regarded at all. The notices in the press of Canada of the death of Dr. Beers seem to regard him from the stand-point of the patriot and athlete, or, as the *Witness* puts it, a "good man who did much for Canada and the Empire."

This, from a national and local view, is the only position that could possibly be taken; but has not Dr. Beers a stronger claim on the dental profession of Canada than he had upon the general public? A monument, not in marble or granite, but more enduring, might be raised as a memorial to him by his profession throughout the Dominion. It is not for this journal to suggest its character, but something might be done to show a professional appreciation of his work.

The Canadian journals have much to say of Dr. Beers's address before the New York State Dental Association, October 25, 1888, and the *Fredericton Gleaner* regards this speech as one that "crushed through the sophistries and delusions of people in the United States and some few in Canada." It is not surprising that this address created an enthusiasm throughout Canada and is now recalled there with pride. It was truly a scathing denunciation of a nation upon its own soil, but then Dr. Beers was nothing if not courageous where his convictions were involved. Unfortunately for the comfort of his auditors, there was a good deal of truth mingled with partisan error in that address, and neither have much force at the present time. While there has not been much advance in the "true grandeur of nations" in the twelve years since that address was delivered, the idea is slowly growing that the detestable motto, "My Country, right or wrong," has fewer adherents than at that period. It is to be hoped that the people of Canada will erect the proposed monument, for by so doing they will honor a man true to his convictions, and where one such is found and lost he is worthy of the most appreciative remembrance.

